# Genetic variability of *FOXP2* and its targets *CNTNAP2* and *PRNP* in frontotemporal dementia: A pilot study in a southern Italian population

**DOI:** 10.1016/j.heliyon.2024.e31624

**Published:** 2024-05-22

**Authors:** Paolina Crocco, Francesco De Rango, Francesco Bruno, Antonio Malvaso, Raffaele Maletta, Amalia C. Bruni, Giuseppe Passarino, Giuseppina Rose, Serena Dato

**Affiliations:** a Department of Biology, Ecology and Earth Sciences, University of Calabria, Rende, Italy; b Regional Neurogenetic Centre, ASP Catanzaro, Lamezia Terme, Italy; c IRCCS Mondino Foundation – National Neurological Institute, Department of Brain and Behavioral Sciences, University of Pavia, Italy

**Keywords:** Frontotemporal dementia, FTD, FOXP2, PRNP, CNTNAP2, SNPs

## Abstract

The Forkhead box P2 (FOXP2) is an evolutionary conserved transcription factor involved in the maintenance of neuronal networks, implicated in language disorders. Some evidence suggests a possible link between *FOXP2* genetic variability and frontotemporal dementia (FTD) pathology and related endophenotypes. To shed light on this issue, we analysed the association between single-nucleotide polymorphisms (SNPs) in *FOXP2* and FTD in 113 patients and 223 healthy controls. In addition, we investigated SNPs in two putative targets of FOXP2, *CNTNAP2*, Contactin-associated protein-like 2 and *PRNP*, prion protein genes. Overall, 27 SNPs were selected by a tagging approach. *FOXP2*-rs17213159-C/T resulted associated with disease risk (OR = 2.16, P = 0.0004), as well as with age at onset and severity of dementia. Other *FOXP2* markers were associated with semantic and phonological fluency scores, cognitive levels (MMSE) and neuropsychological tests. Associations with language, cognitive and brain atrophy measures were found with *CNTNAP2* and *PRNP* genetic variability. Overall, although preliminary, results here presented suggest an influence of regulatory pathways centred on FOXP2 as a molecular background of FTD affecting neurological function of multiple brain areas.

## 1 Introduction

The Forkhead box P2 is a transcription factor encoded by the *FOXP2* gene highly expressed in several brain regions during development and adulthood and currently considered as a major actor in human nervous system evolution and development [1,2]. It was the first gene directly implicated in a rare monogenic speech and language disorder segregating in a large multigenerational pedigree, the so-called “KE” family, with severe language impairment [3]. Since this first report, several genetic alterations of *FOXP2* have been identified in multiple cases of speech and language disorders, both inherited and de novo [4,5], as well as with schizophrenia and autism [6,7], and research into its cellular and neurobiological functions highlighted a plethora of fascinating contributions of this transcription factor to the development and function of the neural circuits relevant for human communication [8,9]. As for pathways, FOXP2 is involved in neurite growth, axon guidance, and synaptic plasticity, which may reflect the fact that the primary function is associated with the development and maintenance of neuronal networks [10].

Speech and language impairments are important clinical manifestations in several neurodegenerative disorders, raising the question as to whether common variants in *FOXP2* might be associated with the pathogenesis and/or the clinical features of these diseases.

Language disturbances represent a core trait in patients with Frontotemporal Dementia (FTD), the second commonest cause of dementia characterized by the progressive degeneration of the frontal and anterior temporal brain regions [11,12]; 30–50% of the frontotemporal forms of dementia are familial and transmitted in an autosomal dominant manner, while the rest are sporadic forms.

Among the broad spectrum of clinical phenotypes of FTD, the behavioural variant is the most common form. It is characterized by disinhibition, inappropriate social comportments, and apathy and may be associated with a language profile including deficits in different aspects of language processing, like naming, comprehension of single words, and semantic processing of verbal stimuli [13]. The language variants (collectively referred to as primary progressive aphasia, PPA) are characterized either by impaired speech production and grammatical errors (non-fluent aphasia, nfvPPA) or by impaired word comprehension and loss of meaning of words and objects (semantic variants of a primary progressive aphasia, svPPA) [14]. The phenotype of language disorder in FTD patients has sparked interest in the scientific community, investigating the hypothesis that genes related to language as *FOXP2* might drive the anatomical distribution of regional atrophy in FTD and clinical presentation.

Given its expression in multiple neural sites, along with the phenotypic complexity of FTD, the relationship between FOXP2 and the diseases is probably complex. Nevertheless, this relationship is supported by some studies. Lopez-Gonzales for example observed *FOXP2* expression abnormalities in sporadic and familial frontotemporal degeneration tauopathies [15]. Furthermore, *FOXP2* genetic variations have been associated with FTD endophenotypes like greater brain atrophy and differential hypoperfusion in the language areas in FTD patient [16,17]. In the study by Padovani, the *FOXP2* common genetic variability was additively associated with verbal fluency tasks [16]. In a subsequent study, the same group found similar results when analysing patients with PPA [18]. In these studies, however, no association between the presence of risk alleles and disease, either FTD or PPA, was detected. Notwithstanding, it would be unwise to reject the possibility that genetic variants of *FOXP2* may confer risk to FTD, beside to connection to the language related symptoms of this disorder. Considering the role of FOXP2 as a transcription factor evolutionarily very conserved, binding to near hundreds of gene promoters in the human genome [2,19], in this study we tested the association of *FOXP2* variability in *FTD* and also investigated the genetic variability of two putative targets of FOXP2, prioritized on the base of their role as important players for speech and language development, like *CNTNAP2* (Contactin-associated protein-like 2) and *PRNP* (prion protein gene) [1,8,20].

*CNTNAP2*, whose role as FOXP2 target has been experimentally demonstrated in animal and cell-based models [10,21,22], encodes CASPR2, a transmembrane protein of the neurexin family widely expressed in the brain and involved in nerve transmission, migration of neurons, neurite prolongation and connectivity [[22], [23]]. In FTD patients, variations in *CNTNAP2* were related to cortical thickness abnormalities in language-related brain areas [17]. Moreover, it was suggested that the FOXP2-CNTNAP2 collaboration may represent a mechanistic link between clinically distinct syndromes involving disrupted language [21].

*PRNP* was chosen because mutations in this gene may be a rare cause in the FTD spectrum [24]; furthermore, variants in this gene have been associated with FTD [25] and indicated as risk factors or disease-modulators in impaired language phenotypes like PPA, in collaboration with markers of *FOXP2* and *APOE* [18]. Moreover, recent literature hypothesizes prion-like mechanisms in neurodegenerative conditions other than prion diseases, such as Alzheimer's disease, Parkinson's disease, motor neuron disease and frontotemporal dementia [26].

The sample analysed comprised individuals affected by sporadic FTD and age-matched healthy controls, both recruited in Southern Italy (Calabria region), an area characterized by high genetic homogeneity because of historical and geographical isolation.

Associations of the genetic variability at selected SNPs in *FOXP2*, *PRNP* and *CNTNAP2* were tested with respect to the disease occurrence, the onset, the cognitive status, and language ability, as well as the possible effect on brain structures. Conditional association analyses were performed, to test the hypothesis that the cooperative expression of *FOXP2* with other genes can have a role in the neurodegeneration leading to the development of human diseases.

## 2 Materials and methods

### 2.1 Participants and data collection

DNA samples from patients were provided by the Regional Neurogenetic Centre (ASP CZ) and were collected from 2006 to 2018. Diagnosis of FTD was carried out according to Lund and Manchester criteria [27,28]. All patients diagnosed with FTD were evaluated with neuropsychological tests. Information was extracted retrospectively from medical records, on the base of clinical data completeness.

Control subjects were recruited in the same population of patients, in several different recruitment campaigns aimed to the monitoring of the quality of aging in Calabria [29]. The health status of all the subjects were carefully assessed, to exclude the presence of any neurological disorder. To avoid potential causes of false associations as population stratification effects, only subjects with at least two generations of ancestors from the Calabria region were included in the sampling.

Samples were excluded in case of: (a) cerebrovascular disorders, previous stroke, hydrocephalus (b) a history of traumatic brain injury or another neurological disease; (c) significant medical problems (e.g., diabetes or hypertension; cancer within the past 5 years; clinically significant hepatic, renal, cardiac, or pulmonary disorders); (d) history of major depressive disorder, bipolar disorder, schizophrenia, substance abuse disorder, or mental retardation. Patients were tested to exclude the presence of mutations in causative FTD genes (*GRN, MAPT, VCP* and *TARDBP*) and *C9ORF72*, which have been reported to be common in the Calabrian region [30,31].

### 2.2 Genetic analysis: SNP selection and genotyping

A total of 27 SNPs, 11 mapping on *FOXP2*, 11 mapping on *CNTNAP2* and 5 mapping on *PRNP*. To identify candidate SNPs, it was carried out a hybrid functional and tagging approach. SNPs with a minor allele frequency (MAF) ≥ 5% in Caucasians (International HapMap Project, version 28; http://www.hapmap.org) were considered in the specific gene regions, including the 5′ and 3’ neighbouring sequences. Tagging SNPs were selected by Haploview Tagger Program (http://www.broad.mit.edu/mpg/haploview/; http://www.broad.mit.edu/mpg/tagger/), using pairwise tagging with a minimum r^2^ of 0.8. Additional SNPs significantly associated with language impairment and analysed in previous studies were selected [17,29,30[32][33]]. After genotyping, from the starting number of markers, four of them were excluded, as reported in Table 1S, one due to a call rate lower than 90% and three because of HWE departure in controls (p < 0.05), leaving a total of 23 SNPs for the following analyses.

**Table 1:** Baseline characteristics of the analysed population, classified as FTD and control group.

	FTD (113)	CONTROLS (223)	*P*-value
Age (years)	69.5 ± 7.64	73.5 ± 5.43	<0.001
Age at onset (years)	68.9 ± 8.5		
Gender (Female, %)	58.4%	51.1%	0.125
*MMSE total score*	14.5 ± 6.95	23.9 ± 3.66	<0.001
Severe impairment: score 0–17	56.0%	6.2%	
Mild impairment: score 18–23	25.3%	32.8%	
Un-impairment: score 24–30	18.7%	61.0%	
*ADL*			<0.001
Severely disabled (ADL = 0–1–2)	19.2%	3.9%	
Moderately disabled (ADL = 3–4)	19.2%	13.5%	
Not disabled (ADL = 5)	61.5%	82.6%	
*CDR*	1.85 ± 1.13		Not available
Non demented (0)	4.2%		
Very mild to mild dementia (0.5–1)	50.0%		
Moderate dementia (2)	45.8%		
*CIRS*	5.04 ± 3.28		Not available
Disease absents or not severe (0–1)	57.9%		
Moderate disability (2)	5.3%		
Severe pathology (3)	5.3%		
Severe disease with requirement of treatment (4)	31.6%		
*NPI Total*	25.1 ± 18.98		Not available
High score (>21)	57.1% (32)*		
Middle scores (11–17)	26.8% (15)*		
Low scores (3–9)	16.1% (9)*		
Phonological fluency	14.02 ± 9.34		Not available
Semantic fluency	6.48 ± 3.77		Not available
Babcock Story	3.95 + 3.62		Not available
Copying drawings task	5.79 + 2.99		Not available
Rey Auditory Verbal Memory Test (Immediate)	23.08 + 9.97		Not available
Rey Auditory Verbal Memory Test (Recall)	3.48 + 2.90		Not available
Corsi Block-Tapping Test	3.82 + 1.24		Not available
Attentive matrices	38.88 + 14.36		Not available

Data are presented as (%) or mean ± standard deviation. MMSE (MiniMental State Examination); ADL (Activity of Daily Living); CDR (Clinical Dementia Rating scale); CIRS (Cumulative Illness Rating Scale); NPI (Neuropsychiatric Inventory, with asterisc is reported the meean value in the specific sub-group). Language ability was measured by verbal fluency tests, typically consisting of two tasks: category fluency (also called semantic fluency) and letter fluency (also called phonemic or phonological fluency). In the standard versions of the tasks, participants are given 1 min to produce as many unique words as possible within a semantic category (category fluency) or starting with a given letter (letter fluency). The participant's score in each task is the number of unique correct words.

Table 1S reports the complete list of the selected SNPs, their position (relative to the chromosome and to the gene), their MAF (Minor Allele Frequency) in Tuscany (Eur). Multiplex SNP genotyping was performed by iPLEX Gold technology (Sequenom Inc, San Diego, USA). Primers for both PCR and single base extension were designed by using designer software (version 3). Multiplex amplification, PCR production purification with the shrimp alkaline phosphatase (SAP) and a primer extension reaction were performed using standard procedures. Then, primer extension products were desalted on resin and spotted on a 384-element SpectroCHIP (Sequenom). Finally, MALDI-TOF analysis was performed by using SpectroACQUIRE v3.3.1.3 (Sequenom). MassARRAY Typer v3.4 Software (Sequenom) was used to analyse spectra. More than 10% of the samples were analysed in duplicate giving a concordance rate of the genotypes calls higher than 99%.

### 2.3 Phenotypic parameters

At first evaluation, each patient underwent a multidimensional assessment including: (i) the Mini Mental State Examination (MMSE) [34] to evaluate the general cognitive status; (ii) the Clinical Dementia Rating scale (CDR), to quantify the severity of symptoms of dementia [35]; (iii) the Activity of Daily Living (ADL) and Instrumental Activity of Daily Living (IADL) to assess the level of impairment of basic and instrumental functioning, respectively [36,37]; (iv) the Clinical Insight Rating Scale (CIRS), to evaluate the awareness of deficits [38] (v) the Neuropsychiatric Inventory (NPI) to assess the presence of neuropsychiatric symptoms [39]. Furthermore, a battery of neuropsychological tests was administered to obtain information about the functionality of different areas of cognition.

These comprise: (i) Phonological and Semantical verbal fluency tests [40,41] to evaluate language abilities; (ii) Rey Auditory Verbal Memory Test (RAVLT) and Babcock Story to assess verbal learning and memory [40,41]; (iii) Corsi Block-Tapping Test to evaluate visuo-spatial short term working memory [40]; (iv) Attentive matrices to assess selective attention and executive functions [42] and (v) a copying drawings task to determine the presence of constructional praxis [41].

A sample of 75 participants underwent brain MRI scan at the time of the diagnosis, and were visually analysed by a trained neurologist, for extracting the rating scores of medial temporal lobe atrophy (MTA), posterior atrophy (PA), global cerebral atrophy frontal sub-scale (GCA-F), and Fazekas scale. According to many authors, visual rating scales represent a suitable method for assessing atrophy in a clinical setting and may aid the differentiation of FTD from AD, as well as AD from normal brain aging [43] and are correlated with disease's duration and severity [44]. Below the description of each scale:

**Medial temporal lobe atrophy:** The MTA scale, also known as Scheltens' scale, allow to assess the degree of atrophyin the hippocampus, parahippocampal gyrus, and surrounding CSF spaces. It ranks from 0 to 4, with grade 0 indicating no cortical atrophy; grade 1, enlargement of choroid fissure; grade 2, enlargement of the temporal horn of the lateral ventricle; grade 3, moderate loss of hippocampal volume; and grade 4, severe loss of hippocampal volume [45].

**Posterior atrophy:** The PA scale is a 4-point scale evaluating posterior cortical atrophy. It ranks from 0 to 3, with grade 0 showing no cortical atrophy; grade 1, mild parietal cortical atrophy, with mild enlargement of the posterior cingulate and parieto-occipital sulcus; grade 2, substantial parietal atrophy, with enlargement of the posterior cingulate and parieto-occipital sulcus; grade 3, end-stage “knife-blade” atrophy, with extreme enlargement of the posterior cingulate and parieto-occipital sulcus [46].

**Global cerebral atrophy-frontal sub-scale:** The GCA-F, also known as Pasquier scale, evaluates cerebral atrophy in the frontal lobe as delimited by the central sulcus. It scores the degree of atrophy from 0 to 3, with grade 0 representing no cortical atrophy; grade 1, mild atrophy (dilatation of sulci); grade 2, moderate atrophy (loss of gyri volume); and grade 3, end-stage “knife blade atrophy” [47].

**Fazekas scale:** The Fazekas scale is used to quantify the amount of white matter lesions. It ranks from 0 to 3, with grade 0 indicating absence of punctate lesions or single punctate lesions (maximum 3); grade 1, presence of multiple (3) punctate lesions; grade 2, lesions which begins to be confluent (bridging); and grade 3, large confluent lesions [48].

Atrophy index was calculated by using a protocol of visual rating scales suggested by Fumagalli et al., 2018, set up for identify patterns of atrophy in key regions of the brain (orbitofrontal, anterior cingulate, frontoinsula, anterior and medial temporal lobes and posterior cortical areas) [49].

### 2.4 Statistical analysis

For each SNP, departure from Hardy–Weinberg equilibrium (HWE) was assessed in controls by X^2^ tests. Logistic regression models were used to estimate how the variability of analysed genes influences the predisposition to FTD. Odds ratios (ORs) and their 95% confidence intervals (CIs) were estimated by using age and gender as covariates in the formulated regression models. For each SNP, additive, dominant, and recessive genetic models were evaluated and the best model was considered the one with a lower p-value. Conditional logistic regression analysis was performed to investigate the independent effect of SNPs on disease occurrence. Linear regression was applied for determining the effect of polymorphisms on phenotype variables. For standardizing the distribution, values were transformed into z-scores, by subtracting the mean and dividing by the standard deviation. As for the MRI measurements, for all rating scales, score distribution was skewed, we reclassified the scores for MTA, PA and GCA-F as dichotomous (0: no atrophy; 1: from mild to severe atrophy) and applied the linear regression ; Fazekas scale were treated as continuous variable and logistic regression applied, adjusting for age and sex.

All statistical analysis was performed using R Statistical Software (version 4.1.2, R Foundation for Statistical Computing, Vienna, Austria) (http://www.R-project.org/) and Plink Version 1.9 (http://cog-genomics.org/plink/1.9/). A *P*-value less than 0.05 was considered statistically significant. False discovery rate (FDR) correction was applied to control for multiple testing, assuming a significance threshold (q-value) of 0.05.

## 3 Results

Table 1 reports the baseline characteristics of the analysed population, composed by 113 FTD patients and 223 samples from the control group. Differences were found for age, MMSE and ADL (p < 0.001). Controls were 4 years older than cases, while cases showed a lower performance of MMSE than healthy controls and with a higher level of disability, as it was expected. The mean age at onset of the disease was 68.9 + 8.5 years of age and the mean disease duration, which is a practical measure of diseases severity, was 4 years (time spent between the first diagnosis and last assessment or death).

### 3.1 Association between polymorphisms and FTD

From the full list of genotyped SNPs, 1 was excluded from the analysis because of a call rate lower than 90%, and 3 because of HWE departure in control populations, leaving a total of 23 SNPs useful for the analysis. Table 2S reports the genotypic frequencies at each SNP passing the quality control, in case and controls. As reported in Table 2, logistic regression analysis, carried out adjusting for age and sex, showed significant differences between FTD and controls for the *FOXP2* variant rs17213159-C/T, both under an additive (OR = 2.16, 95% C.I.:1,41–3,31; *p* = 0.0004) and dominant (OR = 2.19, 95% C.I. 1,30–3,70; *p* = 0.0033) model. This association remains significant also after FDR correction for multiple testing, as reported in Table 2. In support of this result, the same association with the disease was found when a cohort of self-declared healthy Italians (Tuscans), whose genotypes were retrieved from 1000 Genomes Project (www.1000genomes.org, accessed on October 18, 2023) was used as an additional control group [(OR: 4.05; 95% C.I.:2.35–6.95; p < 0.001 under an additive model) (OR: 4.98; C.I.:2.70–9.18; *p* = 0.0002 under a dominant model),].

**Table 2:** Association analysis of *FOXP2*, *CNTNAP2* and *PRNP* SNPs with FTD, obtained by logistic regression.

SNP (Alleles) Gene	OR (95% CI)	*p*-value FDR	
**rs17213159 (C/T) FOXP2**	**2.16 (1.41-3.31)^A^**	0.0004	0.0092
rs10255943 (G/A) FOXP2	1.17 (0.69-1.96)^A^	0.548	0.727
rs1229761 (A/C) FOXP2	1.31 (0.85-2.03)^A^	0.219	0.559
rs4727799 (T/C) FOXP2	0.55 (0.27-1.11)^D^	0.097	0.446
rs7782412 (C/T) FOXP2	0.85 (0.51-1.44)^A^	0.569	0.727
rs1456029 (A/G) FOXP2	0.74 (0.41-1.32)^D^	0.314	0.624
rs7799652 (T/G) FOXP2	1.29 (0.69-2.40)^D^	0.411	0.703
rs2396752 (T/C) FOXP2	0.79 (0.40-1.54)^A^	0.497	0.714
rs17372022 (T/G) FOXP2	0.483 (0.15-1.49)^D^	0.206	0.559
rs10230558 (T/A) FOXP2	0.683 (0.42-1.10)^A^	0.117	0.448
**rs10230373 (A/G) CNTNAP2**	**0.48 (0.24-0.97)**^**D**^	**0.041**	0.414
rs10246256 (T/C) CNTNAP2	0.62 (0.29-1.32)^D^	0.218	0.559
rs1918295 (A/G) CNTNAP2	1.51 (0.68-3.30)^A^	0.302	0.624
rs2710117 (A/T) CNTNAP2	0.90 (0.47-1.70)^A^	0.746	0.746
rs826644 (A/G) CNTNAP2	0.83 (0.52-1.31)^A^	0.428	0.703
rs851715 (T/C) CNTNAP2	0,82 (0.47-1.42)^A^	0.490	0.714
rs2538976 (C/T) CNTNAP2	0.88 (0.44-1.77)^D^	0.729	0.746
rs2373289 (A/T) CNTNAP2	1.21 (0.54-2.70)^D^	0.641	0.746
rs2972106 (G/A) CNTNAP2	1.34 (0.74-2.42)^D^	0.326	0.624
rs6464737 (C/G) CNTNAP2	0.86 (0.46-1.61)^D^	0.649	0.746
rs2756271 (G/A) PRNP	1.51 (0.94-2.40)^A^	0.081	0.446
rs2855412 (A/G) PRNP	1.98 (0.98-3.98)^D^	0.054	0.414
rs13045348 (T/C) PRNP	0.89 (0.52-1.54)^D^	0.694	0.746

^a^ Logistic regression OR adjusted for age and sex.

^b^ For each SNP the best model was considered: A additive, D dominant. Significant *p*‐values (<0.05) are highlighted in bold.

We also found association with the disease for rs10230373-A/G of *CNTNAP2* (OR = 0,48, 95% C.I.: 0,24–0,97; *p* = 0,043) and borderline for rs2855412-A/G in *PRNP* (OR = 1,98, 95% C.I.: 0,98–3,98; *p* = 0,054) under a dominant model. However, these associations do not hold multiple comparisons, as shown in Table 2.

Finally, we performed a conditional logistic regression analysis for testing if the effects of the two nominally significant SNPs (rs17213159 and rs1023073) showed independent effects. The analysis demonstrated that the effects were not independent because the effect of rs1023073 on the disease were not more significant when considered together (p = 0,14). The effect of the *FOXP2* variant rs17213159 on the disease increased when considered together with the second variant, in line with the regulatory effect of FOXP2 on its target (OR_ADD_ = 3,13, 95% C.I. 1.8–5.4; p < 0.001).

### 3.2 Association between polymorphisms and FTD endophenotypes

In the case-only approach, we tested the association with the disease onset and found that only the SNP rs17213159-C/T was nominally associated with the age of onset (β: 2.688, p = 0.046) under a dominant model. Table 3 reports the results of the analysis with MMSE and CDR in the analysed population. A higher MMSE performance was observed in FTD patients carrying at least one copy of the minor allele of rs10255943 and rs2396752 of *FOXP2* (β = 4.692, *p* = 0.030 and *β* = 6.044, *p* = 0.019, respectively). Conversely, MMSE scores were significantly lower in individuals carrying at least one copy of the minor allele of rs4727799 and rs7799652 at *FOXP2* than in those homozygous for the common alleles (*β* = −3.694, *p* = 0.025 and *β* = −2.984, *p* = 0.043, respectively). For what concerns *FOXP2* targets, patients carrying at least a copy of the minor allele of rs2972106-*CNTNAP2* showed lower MMSE scores (*β* = -3.112, *p* = 0.041), while a trend of positive association was observed for the rs2756271 at *PRNP* (*β* = 2.673, *p* = 0.046).

**Table 3:** Significant associations between genetic variability at the different markers of *FOXP2*, *CNTNAP2* and *PRNP* and MMSE and CDR scores in FTD samples, under an additive model.

SNP-GENE	Minor Allele	β	*P*-value
MMSE			
rs10255943-FOXP2	A	4.692	0.030
rs4727799-FOXP2	T	−3.694	0.025
rs2396752-FOXP2	C	6.044	0.019
rs7799652-FOXP2	G	−2.984	0.043
rs2972106-CNTNAP2	A	−3.112	0.041
rs2756271-PRNP	A	2.673	0.046
CDR			
rs17213159-FOXP2	T	−0.925	0.029

*MMSE: Mini Mental State Examination CDR: Clinical Dementia Rating scale.

Associations with dementia severity (CDR) was found for the same marker of *FOXP2* rs17213159 previously associated with the disease risk, with T-carriers associated with a lower CDR value (*β* = −0.925, *p* = 0.029), underlying a protective effect of the minor allele T respect to the severity of dementia.

There was no correlation between selected SNPs with CIRS and NPI total scores. The results of the analysis of association with neuropsychological tests comprising also the language tests are reported in Table 4, where significant associations are represented. As for the language, significant association was found between rs7799652-*FOXP2* variability and both semantic (*β* = −3.594, *p* = 0.033), and phonological fluency scores (*β* = −7.379, *p* = 0.036), with G-carriers showing a lower performance in both tests (median value 4.1 *vs* 8.6 TT for semantic and 9.38 *vs* 20.28 TT for phonological test) with an allelic dosage effect. Significant association with semantic fluency test was found also for the rs851715 at *CNTNAP2 (β* = −4.939, *p* = 0.023), with C-carriers performing worse respect to TT (median value 3.18 vs 8.38 TT).

**Table 4:** SNPs significantly associated with neuropsychological tests.

Test	*rs7799652-FOXP2*	*rs17372022- FOXP2*	*rs1229761- FOXP2*	*rs851715-CNTNAP2*	*rs1918295- CNTNAP2*	*rs2756271-PRNP*
Phonological Fluency	**β = -7.379, *p* = 0.036**	β = 1.027, *p* = 0.898	β = 0.719, *p* = 0.821	β = −1.879, *p* = 0.762	β = −8.877, *p* = 0.064	β = 1.882, *p* = 0.579
Semantic Fluency	**β = -3.594, *p* = 0.033**	β = −4.075, *p* = 0.314	β = −2.025, *p* = 0.230	**β = -4.939, *p* = 0.022**	β = 2.761, *p* = 0.281	β = 1.600, *p* = 0.476
Babcock Story	**β = -4.810, *p* = 0.0002**	**β = -6.237, *p* = 0.045**	**β = -3.160, *p* = 0.037**	β = 2.176, *p* = 0.426	β = −1.198, *p* = 0.683	β = −0.118, *p* = 0.920
Copying drawings task	β = −1.409, *p* = 0.163	β = 1.281, *p* = 0.542	β = −0.149, *p* = 0.860	β = 0.151, *p* = 0.924	β = 1.137, *p* = 0.412	**β = 1.770, *p* = 0.019**
Rey Auditory Verbal Memory Test (Immediate)	β = −1.387, *p* = 0.698	**β = -18.99, *p* = 0.003**	β = 0.752, *p* = 0.813	β = 2.748, *p* = 0.583	β = 3.206, *p* = 0.558	β = −2.276, *p* = 0.480
Rey Auditory Verbal Memory Test (Recall)	β = −0.769, *p* = 0.440	**β = -4.155, *p* = 0.050**	β = −0.532, *p* = 0.583	β = 0.036, *p* = 0.981	β = 0.729, *p* = 0.670	β = −1.361, *p* = 0.120
Corsi Block-Tapping Test	β = −0.461, *p* = 0. 232	β = −0.842, *p* = 0.380	β = −0.085, *p* = 0.861	β = −1.043, *p* = 0.292	β = 1.014, *p* = 0.140	**β = 0.762, *p* = 0.027**
Attentive matrices	β = −1.765, *p* = 0.722	β = 1.131, *p* = 0.903	β = 0.080, *p* = 0.986	β = −12.28, *p* = 0.160	**β = 21.85, *p* = 0.015**	β = −0.790, *p* = 0.867

N.B: Additive model was considered for all the analyses.

Consistently with the previous association of rs7799652-*FOXP2* with MMSE and verbal fluency, we found that G-carriers at rs7799652 of *FOXP2* performed worse at Babcock Story test (*β* = −4.810, *p* = 0.0002). The same test was found associated to other *FOXP2* markers as rs17372022, with G-carriers showing lower values of the test (*p* = 0.045) and rs1229761, with C-carriers showing a lower performance in the test (*p* = 0.037). rs17372022-*FOXP2* was found associated also with Rey Auditory Verbal Memory Test both immediate (*p* = 0.003) and recall (*p* = 0.050), although the last online borderline.

SNPs in the targets were associated with some neuropsychological tests in FTD patients. rs1918295 in *CNTNAP2* was found associated with Attentive matrices (*β* = 21.85, *p* = 0.015) while rs2756271 in *PRNP* was found associated with both copying drawings and Corsi Block-Tapping Test: in both cases A-carriers showed a higher performance to the tests respect to GG genotype (median value 6.6 *vs 3.*3 GG in the first test, 0.35 *vs* 0.29 in the second test).

### 3.3 Association between polymorphisms and multiple visual rating scales in FTD patients

To the aim of identifying if different and typical patterns of brain atrophy were present in relation to a particular genotype at the analysed genetic markers, we applied linear and logistic regression analyses to the MTA (Medial temporal lobe atrophy), PA (Posterior atrophy), GCA-F (Global cerebral atrophy-frontal sub-scale) and Fazekas rating scores, retrieved by analysing brain sMRI scan collected at the time of the diagnosis, as described in the Materials and methods section. Data on imaging were available for 75 FTD patients and Table 5 resumes the results of associations. Significant associations at 5% nominal level were found for rs13045348-*PRNP* with PA (*p* = 0.022) and rs2972106-CNTNAP2 with Fazekas scale (*p* = 0.038). In both cases, carriers of at least one copy of the minor allele T showing higher values of brain atrophy and a higher number of lesions. rs17213159-FOXP2 reported not significant associations (p values close to 0.080) which can depend on the sample size and may only delineate trends. Also in this case, carriers of the minor allele exhibit higher degree of brain atrophy.

**Table 5:** Significant associations between SNPs and measures of brain atrophy.

	Medial temporal lobe atrophy MTA	Posterior atrophy PA	Global cerebral atrophy-frontal GCA-F	Fazekas_scale
	*β* (*p*-value)	*β* (*p*-value)	β (*p*-value)	OR [C.I. 95%]; *p-*value
rs17213159-FOXP2	0.534 (0.083)	0.577 (0.077)	0.490 (0.079)	1.56 [0.22–10.95]; 0.653
rs10230373-CNTNAP2	−0.821 (0.090)	0.117 (0.811)	−0.168 (0.727)	–
rs826644-CNTNAP2	−0.088 (0.838)	0.271 (0.498)	−0.053 (0.895)	11.86 [0.68–20.4]; 0.088
rs13045348-PRNP	−0.268 (0.567)	**0.985 (0.022)**	0.443 (0.295)	0.82 [0.09–7.05]; 0.861
rs2972106-PRNP	0.003 (0.994)	−0.256 (0.556)	−0.034 (0.935)	**13.88 [1.15**–**16.74]; 0.038**

MTA: Medial temporal lobe atrophy; PA: Posterior atrophy; GCA-F: Global cerebral atrophy-frontal sub-scale; β (*p*-value) refers to linear regression analysis; OR (C.I. 95%) *p*-value refers to logistic regression analysis. In both cases the most significant genetic model was the dominant.

## 4 Discussion

Aim of this paper was to shed light on the genetic predisposition in FTD, a complex and heterogenous disease, with a common underlying neurodegenerative background, that is the loss of nerve cells in the frontal and temporal lobes of the brain. We focused our attention on *FOXP2* gene, very well known as a crucial player in nervous system evolution and language development from humans to songbirds [50], considering that language impairment is a FTD's endophenotype [17]. Although the role of FOXP2 has been reported in the literature, both physiologically as a molecular entry point in mechanisms that enable human spoken language [8], and pathologically, as it is involved in abnormalities in phonological and linguistic production and in severe speech and language disorders [51], few authors have investigated *FOXP2* as a genetic risk factor for neurodegenerative traits [15,52]. Instead, several reports proposed that both mutations [53] and common variants [54] at *FOXP2* locus may have detectable effects on the brain structure and function, most notably in the inferior frontal gyrus, caudate nucleus, and cerebellum, igniting curiosity about the possible involvement of mutations with a role in the early development of the brain in late-life neurodegenerative diseases. Because *FOXP2's* neuronal expression patterns are dynamic and likely controlled by several factors (upstream regulators, downstream targets and protein-protein interactions), in this study we selected SNPs in two *FOXP2* targets, *CNTNAP2* and *PRNP,* prioritized basically from evidence reporting their effect on language development or speech and language diseases [8, 19, 16, 24, 17] and tested the single-SNP association both with FTD occurrence and onset and with its endophenotypes.

Our results support an influence of *FOXP2* on FTD susceptibility, through the association between the genetic variability at the intronic variant rs17213159-C/T and the disease. This association holds multiple comparison correction and suggests a disadvantageous role of the rarer allele T conferring a major risk of disease. Less influence on disease's risk was found for the genetic variability at the two chosen targets *CNTNAP2* and *PRNP*, with the marker rs10230373-A/G at *CNTNAP2* significantly associated with the disease and only borderline the rs2855412-A/G for the second gene. The effect of the two most significant SNPs is not independent, with the risk related to SNP pairs prevalently due to the *FOXP2* variant, in agreement with the central role of *FOXP2* as regulator of downstream targets. Interestingly, the SNP rs17213159-C/T was found nominally associated with the age of disease onset and severity of dementia, and borderline associated with three out of four measures of brain atrophy, in accordance with a possible role of *FOXP2* in influencing the loss of neurons in different areas. Bioinformatic evidence obtained by eQTL data retrieved by LIBD (https://eqtl.brainseq.org/phase2/eqtl) report that although its intronic location, the polymorphism rs17213159 can be functional, with the risky allele T significantly associated with increased *FOXP2* expression both in hyppocampus and in brain cortex (*p* < 0.0004). Thus, alterations in the levels of this gene might be the molecular basis of the observed associations, and this deserves further attention. In accordance with the driven hypothesis of a possible effect of *FOXP2* genetic variations on language assessment and with the study of Padovani et al., 2010, we found association of *FOXP2* variability with both semantic and phonological fluency scores, with rs7799652 G-carriers showing a lower performance in both tests. The genetic variability of *FOXP2* was found associated with cognitive levels in FTD samples, with both positive (rs10255943 and rs2396752) and negative (rs4727799 and rs7799652) effects on MMSE scores. Other *FOXP2* markers like rs17372022 and rs1229761 were found associated with tests depicting the neuropsychological status of FTD patients, like Rey Auditory Verbal Memory Test immediate and recall (correlated to the variability of rs17372022) or Babcock Story test (significantly associated to rs7799652, rs17372022, rs1229761 variability). The association of the gene with these tests is consistent because all of them reflects verbal abilities. Moreover, a poor performance to Rey's immediate and delayed recall in FTD was related to atrophy of the right dorsolateral prefrontal cortex [55]. As it concerns *FOXP2* targets here analysed, although their genetic variability was only borderline associated with the disease status, this study suggests an influence of their variability on a broader spectrum of disease endophenotypes of FTD.

*CNTNAP2* genetic variability was found associated with semantic fluency, with rs851715 C-carriers performing worse respect to TT. This result agrees with the association of variants of *CNTNAP2* associated with language impairment observed by Whalley et al., 2011 [56], although mediated by different SNPs (rs7794745 and rs2710102), the first one excluded from the present study because of HWE departure in controls in our population. *CNTNAP2* variability was correlated with MMSE scores (rs2972106), attentive matrix test (rs1918295) and white matter lesions (rs2972106 with Fazekas scale). Attentive matrix measures selective attention and executive functions related to the activity in the parietal and frontal areas [57]; furthermore, the association here found is in line with findings demonstrating a higher *CNTNAP2* expression in the frontal and temporal lobes, responsible for language abilities [58[58] and references therein].

*PRNP* variability instead was not found related to language in this sample, but associated with MMSE (rs2756271), copying drawings task and Corsi Block-Tapping test (rs2756271), as well as posterior brain atrophy (rs13045348 with PA). Corsi Block-Tapping test is normally associated with integrity of right posterior parietal cortex and right dorsolateral prefrontal cortex, two parts of a broader brain network involved in the control of cognitive functions such as working-memory, spatial attention, and decision-making [59]. Data on regional *PRNP* expression in the brain support these observations, indicating its presence in different areas in neocortical regions, including frontal cortex, cerebellum, and hippocampus [60].

Limitations: Possible drawbacks to this study can be found. Firstly, a limitation is the small sample size. However, we want to underline that both patients and controls have been collected in Calabria (Southern Italy), a region with a high level of genetic homogeneity due to historical and geographical isolation until recently. Moreover, special attention was paid to the recruitment to avoid false-positive associations due to population stratification. The second drawback is that unfortunately we have no reliable information on learning disabilities in these patients, in part because the diagnosis of learning disabilities is relatively recently recognized, and in part because we analysed a cohort of FTD patients mainly represented by the behavioural variant of frontotemporal dementia (69% of the patients analysed in this paper), known also as frontal variant, mainly characterized by cognitive, personality and social impairment, while the FTD spectrum include another major syndrome, namely primary progressive aphasia (PPA), in which is predominant the characteristic of language disorder. We have a very few patients diagnosed as PPA (9% of the patients) and their statistical power is not sufficient to draw any conclusions about genetic associations with the disease. However, a high frequency of neurodevelopmental learning disability, including dyslexia, has been reported in FTD patients and their first-degree relatives [17], so we argued for the involvement of specific genes potentially causative of susceptibility in language-associated brain regions in FTD. The third drawback can be represented by the fact that it was not the same SNP or couple of SNPs associated with all the tests, but associations were multiple and with different variants, not in linkage disequilibrium among them. This may probably suggest that disease association should be considered at the gene level, and single associations should be investigated in a larger sample of patients and controls, for replicating the suggestive trends here observed.

In conclusion, we may say that the study here presented suggests a role of regulatory pathways centred on *FOXP2* as a molecular background of FTD and possibly in other neurodegenerative diseases. FOXP2 effects can involve the interplay with its targets and affect neurological function of multiple brain areas. The skewed associations here documented may certainly be due to the phenotypic variability of the disease, as well as to the high complexity in the genetic interactions between *FOXP2* locus and its regulators. Suggestive trends here reported should be tested in future multicentred studies collecting different clinical phenotypes described under the FTD spectrum, to test the association of *FOXP2* and its targets as a risk factor for each of the FTD clinical variants. Furthermore, for considering the population-specificity, the observed associations should be replicated in samples of different ethnicity. Hopefully, associations here reported may represent a starting point for new studies on the complexity in the molecular roles of FOXP2 in the brain, and ultimately its effects on brain injuries, beside its role in language impairments, to finally define the contribution of such locus to the genetic susceptibility of FTD, which still represents a major challenge in FTD research.

## Funding

This research was supported by “SI.F.I.PA.CRO.DE.–Sviluppo e industrializzazione farmaci innovativi per terapia molecolare personalizzata PA. CRO.DE.” PON ARS01_00568 granted by MIUR (Ministry of Education, University and Research) Italy to G.P.

## Ethics statement

The study was performed according to the Helsinki Declaration of 1975. Ethical review and approval were not required because the study involves the secondary use of non-identifiable information previously collected and anonymous biological materials, in accordance with the local legislation. The patients/participants provided their written informed consent to participate in this study.

## Data availability statement

The data pertaining to this study will be made available on request to the corresponding author.

## CRediT authorship contribution statement

**Paolina Crocco:** Writing – review & editing, Methodology. **Francesco De Rango:** Formal analysis, Data curation. **Francesco Bruno:** Writing – review & editing, Data curation. **Antonio Malvaso:** Methodology, Data curation. **Raffaele Maletta:** Data curation. **Amalia C. Bruni:** Writing – review & editing. **Giuseppe Passarino:** Writing – review & editing, Funding acquisition. **Giuseppina Rose:** Writing – review & editing, Supervision. **Serena Dato:** Writing – original draft, Supervision, Conceptualization.

## Declaration of competing Interest

The authors declare no conflict of interest.

## Appendix A Supplementary data

Table 1S_resubmission_V2.docx Table 1S_resubmission_V2.docx

Table 2S_V2.docx Table 2S_V2.docx
