# Supplementary material for: Genetic variability of FOXP2 and its targets CNTNAP2 and PRNP in frontotemporal dementia: A pilot study in a southern Italian population
Source: Heliyon. 2024 May 22;10(11):e31624. doi: 10.1016/j.heliyon.2024.e31624 (PMC11140708; doi:10.1016/j.heliyon.2024.e31624)
Supplement: Table 1S_resubmission_V2.docx [file mmc1.docx]

Table 1S.  Description of the 27 selected SNPs.

| **Gene** | **Chr** | **SNP** | **Position (GRCh38.p14)** | **Mutation type (Ensembl)** | **Major/Minor allele (Ensembl-TSI)** | **MAF** |
| --- | --- | --- | --- | --- | --- | --- |
| *FOXP2* | 7 | *rs7795397^b^* | [*114407085*](https://www.ensembl.org/Homo_sapiens/Location/View?contigviewbottom=variation_feature_variation%3Dnormal%2Cseq%3Dnormal;db=core;r=7:114407035-114407135;source=dbSNP;v=rs7795397;vdb=variation;vf=731229270) | *intronic variant* | *T/A* | *0.38* |
|  |  | rs10255943 | [114432388](https://www.ensembl.org/Homo_sapiens/Location/View?contigviewbottom=variation_feature_variation%3Dnormal%2Cseq%3Dnormal;db=core;r=7:114432338-114432438;source=dbSNP;v=rs10255943;vdb=variation;vf=731736857) | intronic variant | G/A | 0.44 |
|  |  | rs4727799 | [114470513](https://www.ensembl.org/Homo_sapiens/Location/View?contigviewbottom=variation_feature_variation%3Dnormal%2Cseq%3Dnormal;db=core;r=7:114470463-114470563;source=dbSNP;v=rs4727799;vdb=variation;vf=730539080) | intronic variant | T/C | 0.49 |
|  |  | rs2396752 | [114478057](https://www.ensembl.org/Homo_sapiens/Location/View?contigviewbottom=variation_feature_variation%3Dnormal%2Cseq%3Dnormal;db=core;r=7:114478007-114478107;source=dbSNP;v=rs2396752;vdb=variation;vf=729843551) | intronic variant | T/C | 0.13 |
|  |  | rs17372022 | [114479880](https://www.ensembl.org/Homo_sapiens/Location/View?contigviewbottom=variation_feature_variation%3Dnormal%2Cseq%3Dnormal;db=core;r=7:114479830-114479930;source=dbSNP;v=rs17372022;vdb=variation;vf=733111910) | intronic variant | T/G | 0.20 |
|  |  | rs1229761 | [114583668](https://www.ensembl.org/Homo_sapiens/Location/View?contigviewbottom=variation_feature_variation%3Dnormal%2Cseq%3Dnormal;db=core;r=7:114583618-114583718;source=dbSNP;v=rs1229761;vdb=variation;vf=729339765) | intronic variant | A/C | 0.45 |
|  |  | rs10230558 | [114605694](https://www.ensembl.org/Homo_sapiens/Location/View?contigviewbottom=variation_feature_variation%3Dnormal%2Cseq%3Dnormal;db=core;r=7:114605644-114605744;source=dbSNP;v=rs10230558;vdb=variation;vf=731539505) | intronic variant | T/A | 0.46 |
|  |  | rs7782412 | [114650360](https://www.ensembl.org/Homo_sapiens/Location/View?contigviewbottom=variation_feature_variation%3Dnormal%2Cseq%3Dnormal;db=core;r=7:114650310-114650410;source=dbSNP;v=rs7782412;vdb=variation;vf=731118150) | intronic variant | C/T | 0.44 |
|  |  | rs7799652 | [114650428](https://www.ensembl.org/Homo_sapiens/Location/View?contigviewbottom=variation_feature_variation%3Dnormal%2Cseq%3Dnormal;db=core;r=7:114650378-114650478;source=dbSNP;v=rs7799652;vdb=variation;vf=731266072) | intronic variant | T/G | 0.05 |
|  |  | rs1456029 | [114650693](https://www.ensembl.org/Homo_sapiens/Location/View?contigviewbottom=variation_feature_variation%3Dnormal%2Cseq%3Dnormal;db=core;r=7:114650643-114650743;source=dbSNP;v=rs1456029;vdb=variation;vf=729393408) | intronic variant | A/G | 0.27 |
|  |  | rs17213159 | [114658653](https://www.ensembl.org/Homo_sapiens/Location/View?contigviewbottom=variation_feature_variation%3Dnormal%2Cseq%3Dnormal;db=core;r=7:114658603-114658703;source=dbSNP;v=rs17213159;vdb=variation;vf=733092214) | intronic variant | C/T | 0.13 |
| *CNTNAP2* | 7 | rs6464737 | 146206562 | intronic variant | C/G | 0.24 |
|  |  | *rs7794745^b^* | [*146792514*](https://www.ensembl.org/Homo_sapiens/Location/View?contigviewbottom=variation_feature_variation%3Dnormal%2Cseq%3Dnormal;db=core;r=7:146792464-146792564;source=dbSNP;v=rs7794745;vdb=variation;vf=731223350) | *intronic variant* | *A/T* | *0.32* |
|  |  | rs826644 | 147432126 | intronic variant | T/G | 0.24 |
|  |  | rs851715 | [147829814](https://www.ensembl.org/Homo_sapiens/Location/View?contigviewbottom=variation_feature_variation%3Dnormal%2Cseq%3Dnormal;db=core;r=7:147829764-147829864;source=dbSNP;v=rs851715;vdb=variation;vf=729166408) | intronic variant | T/C | 0.22 |
|  |  | rs10246256 | 147857715 | intronic variant | T/C | 0.22 |
|  |  | rs2538976 | [147888727](https://www.ensembl.org/Homo_sapiens/Location/View?contigviewbottom=variation_feature_variation%3Dnormal%2Cseq%3Dnormal;db=core;r=7:147888677-147888777;source=dbSNP;v=rs2538976;vdb=variation;vf=729927425) | intronic variant | C/T | 0.45 |
|  |  | rs2710117 | [147904680](https://www.ensembl.org/Homo_sapiens/Location/View?contigviewbottom=variation_feature_variation%3Dnormal%2Cseq%3Dnormal;db=core;r=7:147904630-147904730;source=dbSNP;v=rs2710117;vdb=variation;vf=730002030) | intronic variant | A/T | 0.28 |
|  |  | rs2373289 | [148015217](https://www.ensembl.org/Homo_sapiens/Location/View?contigviewbottom=variation_feature_variation%3Dnormal%2Cseq%3Dnormal;db=core;r=7:148015167-148015267;source=dbSNP;v=rs2373289;vdb=variation;vf=729819763) | intronic variant | A/T | 0.22 |
|  |  | rs2972106 | 148182465 | intronic variant | G/A | 0.32 |
|  |  | rs10230373 | 148265638 | intronic variant | A/G | 0.18 |
|  |  | rs1918295 | 148327116 | intronic variant | A/G | 0.23 |
| *PRNP* | 20 | rs2756271 | [4684616](https://www.ensembl.org/Homo_sapiens/Location/View?contigviewbottom=variation_feature_variation%3Dnormal%2Cseq%3Dnormal;db=core;r=20:4684566-4684666;source=dbSNP;v=rs2756271;vdb=variation;vf=177076443) | promoter region variant | G/A | 0.45 |
|  |  | rs13045348 | [4694943](https://www.ensembl.org/Homo_sapiens/Location/View?contigviewbottom=variation_feature_variation%3Dnormal%2Cseq%3Dnormal;db=core;r=20:4694893-4694993;source=dbSNP;v=rs13045348;vdb=variation;vf=179027401) | intronic variant | T/C | 0.25 |
|  |  | *rs12625444^a^* | [*4701861*](https://www.ensembl.org/Homo_sapiens/Location/View?contigviewbottom=variation_feature_variation%3Dnormal%2Cseq%3Dnormal;db=core;r=20:4701811-4701911;source=dbSNP;v=rs12625444;vdb=variation;vf=178959572) | *intergenic variant* | *C/T* | *0.12* |
|  |  | *rs2181021^b^* | [*4702550*](https://www.ensembl.org/Homo_sapiens/Location/View?contigviewbottom=variation_feature_variation%3Dnormal%2Cseq%3Dnormal;db=core;r=20:4702500-4702600;source=dbSNP;v=rs2181021;vdb=variation;vf=176901707) | *intergenic variant* | *T/A* | *0.29* |
|  |  | rs2855412 | [4704072](https://www.ensembl.org/Homo_sapiens/Location/View?contigviewbottom=variation_feature_variation%3Dnormal%2Cseq%3Dnormal;db=core;r=20:4704022-4704122;source=dbSNP;v=rs2855412;vdb=variation;vf=177081875) | regulatory region variant | A/G | 0.11 |

NB: a) SNPs excluded because of a call rate <90%; b) SNPs excluded because of HWE departure in controls (p<0.05). Minor Allele Frequencies are relative to the Tuscany population (Eur) in Ensembl.org.
