# Supplementary material for: Genetic variability of FOXP2 and its targets CNTNAP2 and PRNP in frontotemporal dementia: A pilot study in a southern Italian population
Source: Heliyon. 2024 May 22;10(11):e31624. doi: 10.1016/j.heliyon.2024.e31624 (PMC11140708; doi:10.1016/j.heliyon.2024.e31624)
Supplement: Table 2S_V2.docx [file mmc2.docx]

Table 2S.  Genotypic frequencies (%) of the SNPs analyzed in the manuscript

| **SNP** | **FTD** | | | **Controls** | | |
| --- | --- | --- | --- | --- | --- | --- |
| **FOXP2** | MM | Mm | mm | MM | Mm | mm |
| rs17213159 (C/T) | 38.7 | 49.5 | 11.8 | 57.9 | 38.8 | 3.3 |
| rs10255943 (G/A) | 45.5 | 37.5 | 17.0 | 41.6 | 36.1 | 22.3 |
| rs1229761 (A/C) | 24.3 | 41.7 | 34.0 | 32.1 | 42.9 | 25.0 |
| rs4727799 (T/C) | 27.4 | 43.4 | 29.2 | 41.2 | 37.2 | 21.6 |
| rs7782412 (C/T) | 31.5 | 46.0 | 22.5 | 37.5 | 42.5 | 20.0 |
| rs1456029 (A/G) | 47.2 | 45.9 | 6.9 | 52.2 | 33.3 | 14.5 |
| rs7799652 (T/G) | 42.6 | 41.9 | 15.5 | 38.5 | 46.1 | 15.4 |
| rs2396752 (T/C) | 75.6 | 20.2 | 4.2 | 73.2 | 26.8 | 0.0 |
| rs17372022 (T/G) | 90.5 | 9.5 | 0.0 | 85.3 | 14.7 | 0.0 |
| rs10230558 (T/A) | 53.6 | 35.7 | 10.7 | 40.5 | 41.4 | 18.1 |
| **CNTNAP2** |  |  |  |  |  |  |
| rs10230373 (A/G) | 78.8 | 18.2 | 3.0 | 64.2 | 31.1 | 4.7 |
| rs10246256 (T/C) | 69.4 | 26.5 | 4.1 | 59.0 | 35.0 | 6.0 |
| rs1918295 (A/G) | 70.7 | 26.9 | 2.4 | 80.0 | 18.9 | 0.1 |
| rs2710117 (A/T) | 66.6 | 28.6 | 4.8 | 67.0 | 25.8 | 1.2 |
| rs826644 (A/G) | 46.9 | 45.3 | 7.8 | 46.8 | 39.8 | 13.2 |
| rs851715 (T/C) | 67.8 | 26.8 | 5.4 | 62.5 | 30.8 | 6.7 |
| rs2538976 (C/T) | 37.7 | 43.4 | 18.9 | 35.7 | 45.5 | 18.8 |
| rs2373289 (A/T) | 74.0 | 24.0 | 2.0 | 74.8 | 22.5 | 2.7 |
| rs2972106 (G/A) | 46.4 | 46.4 | 7.2 | 51.9 | 37.9 | 10.2 |
| rs6464737 (C/G) | 35.8 | 50.0 | 14.1 | 35.2 | 52.6 | 12.2 |
| **PRNP** |  |  |  |  |  |  |
| rs2756271 (G/A) | 16.5 | 33.0 | 15.5 | 21.2 | 32.1 | 8.7 |
| rs2855412 (A/G) | 64.7 | 30.7 | 4.6 | 76.9 | 19.4 | 3.7 |
| rs13045348 (T/C) | 47.4 | 42.1 | 10.5 | 48.1 | 43.9 | 8.0 |

*M : wild type allele, m: mutated allele
